# Spontaneous Pneumomediastinum as a Consequence of Severe Vomiting in Diabetic Ketoacidosis

**DOI:** 10.7759/cureus.2562

**Published:** 2018-05-01

**Authors:** Patricia Guzman Rojas, Jeremy Agostinho, Rami Hanna, Olga Karasik

**Affiliations:** 1 Internal Medicine, University of Central Florida College of Medicine, Orlando, USA

**Keywords:** pneumomediastinum, diabetic ketoacidosis, vomiting

## Abstract

Spontaneous pneumomediastinum (SPM) is a rare entity with a reported incidence of approximately 1:7,000 to 1:100,000 of hospital admissions. It has been described as a complication of various conditions related to increased intrathoracic pressure, like recurrent vomiting, post-partum state, vigorous coughing or sneezing, and others.

We present a 25-year-old man who came in with intractable vomiting, secondary to diabetic ketoacidosis (DKA), and was found to have pneumomediastinum on chest imaging. The patient was treated conservatively, eventually recovering and being discharged after several days.

SPM is rarely seen as a complication of DKA. Diagnosis and management may present a challenge. Usually, the condition is benign and treated conservatively. We report this case to increase awareness among physicians of possible pneumomediastinum in a young male presenting with DKA and dyspnea and to emphasize the benign course of this condition.

## Introduction

Spontaneous pneumomediastinum (SPM) is defined as a pneumomediastinum that is not related to trauma, surgery, or other medical procedures. This entity is usually precipitated by high intrathoracic pressure conditions, such as coughing spells, vomiting, labor, or sneezing (Valsalva maneuvers) [1-5]. Some predisposing factors increase the risk of developing SPM, including smoking, asthma, interstitial lung disease, and others.

It is thought that alveolar barotrauma may lead to an air leak into the pulmonary interstitium or pleural space, resulting in a subcutaneous emphysema of the neck, pneumomediastinum (from air dissecting through the bronchovascular bundles into the mediastinum), pneumothorax, or pneumopericardium.

SPM as a complication of diabetic ketoacidosis (DKA) is very rare and is likely related to vomiting. The following case illustrates a diagnostic and management challenge in a patient presenting with severe DKA, complicated by the unexpected finding of pneumomediastinum.

## Case presentation

A 25-year-old man with no past medical history presented to the emergency department (ED) with two days of intractable vomiting, increasing confusion, and progressive difficulty breathing. The patient denied tobacco or recreational drug use.

Upon arrival to the ED, vital signs were within normal range with a blood pressure of 123/61 mmHg, a temperature of 36.8 degree Celsius, a heart rate of 92 beats per minute, and a respiratory rate of 19 respirations per minute. The physical exam showed an obese, lethargic patient, responsive to verbal stimuli. The lung, heart, and abdomen exam were unremarkable and there was no neck crepitus noted. The laboratory evaluation showed leukocytosis of 25.96 x 1000/mm3, elevated creatinine of 2.17 mg/dL, hyperglycemia of 836 mg/dL, hyponatremia of 128 mEq/L, hyperkalemia of 5.2 mEq/L, and bicarbonate of 5 mEq/L. The anion gap was 25 and a venous blood gas showed a pH of 6.91. Urinalysis was positive for 2+ ketones and 1+ protein.

**Figure 1:**
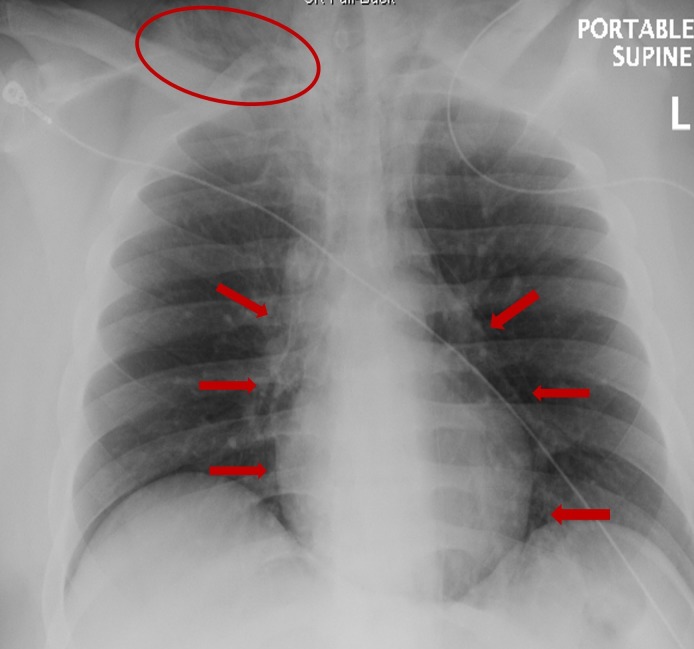
Chest x-rays: pneumomediastinum and subcutaneous emphysema at the right base of the neck Circle: emphysema; Arrows: pneumomediastinum.

**Figure 2:**
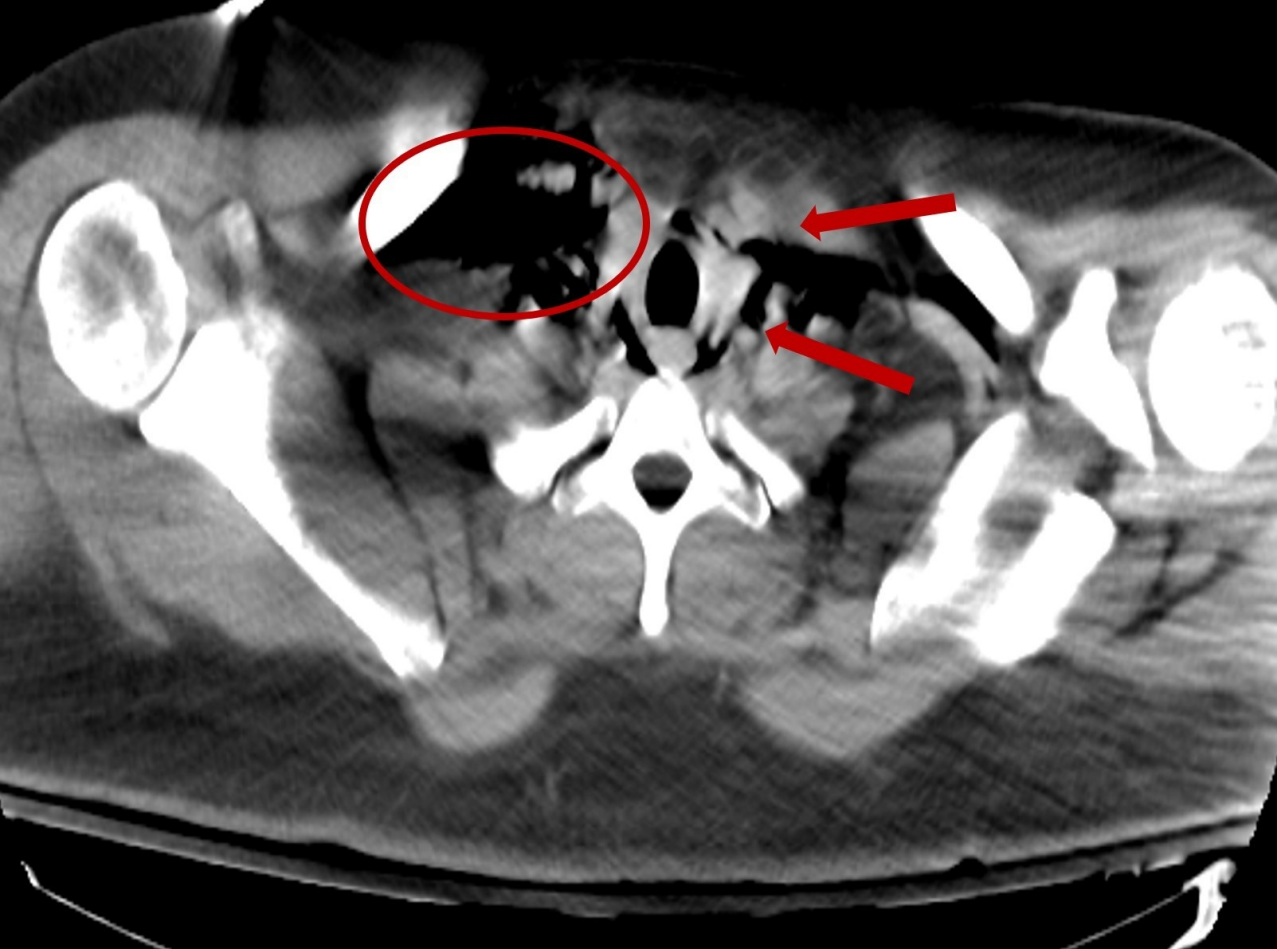
Computed tomography - axial view: extensive pneumomediastinum and emphysema Circle: emphysema; Arrows: pneumomediastinum.

**Figure 3:**
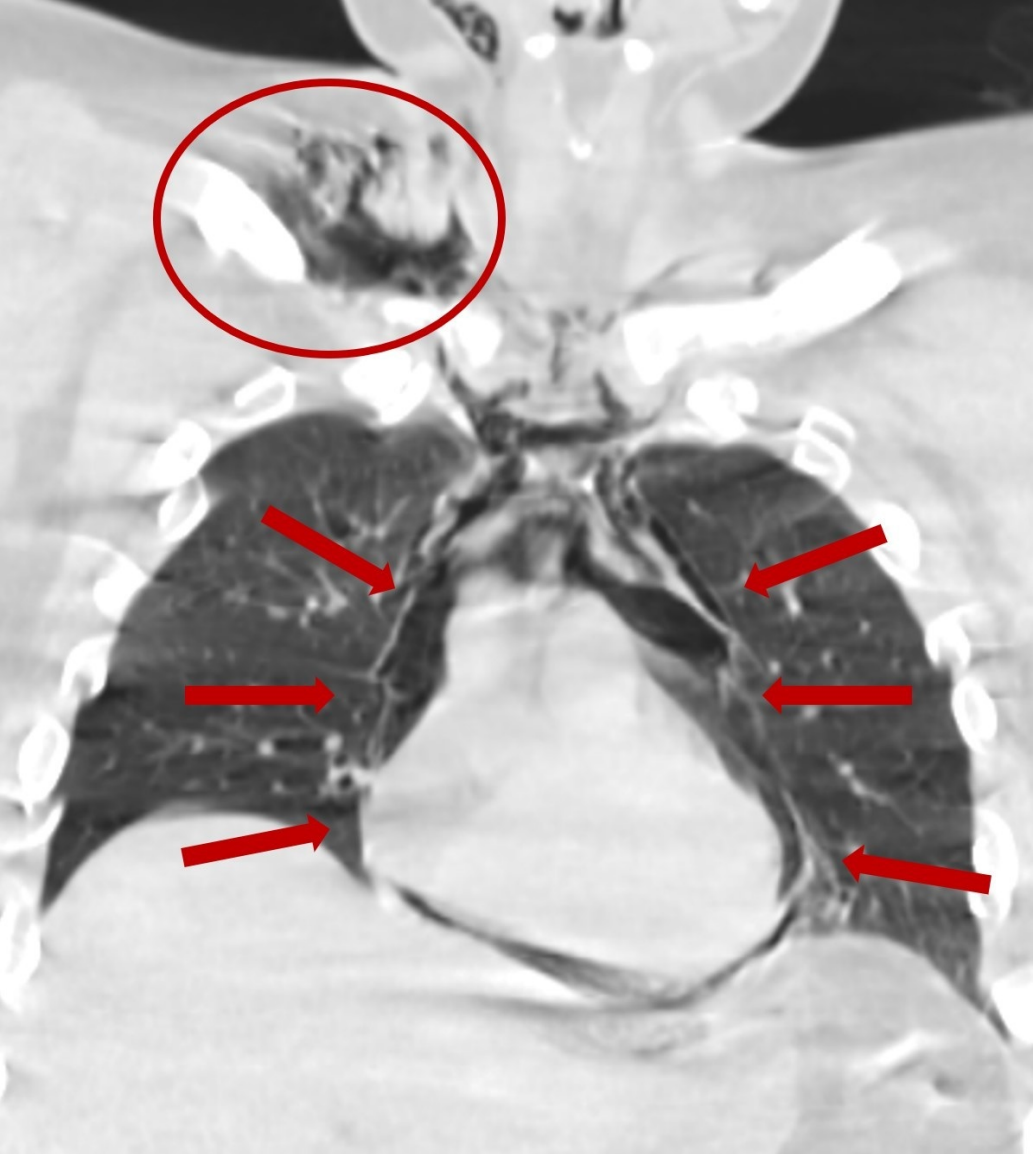
Computed tomography - coronal view: evidence of extensive pneumomediastinum involving the base of the neck, around the great vessels, around the esophagus, and around the pericardium Circle: emphysema; Arrows: pneumomediastinum.

The patient was admitted to the intensive care unit with a new diagnosis of diabetes mellitus complicated with DKA. Intravenous normal saline, bicarbonate, and insulin drip were initiated. The chest X-ray on admission suggested findings compatible with pneumomediastinum (Figure 1). Chest computed tomography (CT) was obtained and reaffirmed the findings (Figures 2-3). Pneumomediastinum in the setting of intractable vomiting was worrisome for esophageal rupture and since our facility was not staffed to manage this clinical entity, the patient was emergently transferred to another facility. The patient was treated conservatively with analgesia and respiratory support and was discharged several days later without any complications.

## Discussion

Pneumomediastinum caused by trauma was first described in 1819. Later, in 1937, Hamman described SPM for the first time. Since that date, only a few case reports have described the association with DKA. SPM is a rare, mostly benign condition, with a reported incidence of approximately 1:7,000 to 1:100,000 among hospital admissions [6]. Comparable to our patient, there is a predominance of the male gender (3:1) with an average age of 20 years [7]. High intrathoracic pressure is usually a precipitating factor of SPM. In our patient, DKA-associated vomiting may have increased alveolar pressure by 20-30 mmHg, which is sufficient to cause alveolar rupture [5].

SPM can be asymptomatic; however, chest pain (most common presentation), shortness of breath, “hot potato voice” (if air accumulates in the pharynx/larynx), and subcutaneous emphysema (in up to 70% of patients) have been reported. Hamman’s sign is an infrequent clinical sign in the presence of mediastinal emphysema and is heard as a crunching sound over the precordium, synchronous with the heartbeat [8-9]. Our patient initially did not present with emphysema on physical exam, but, later, emphysema was palpable in the right cervical area. The initial diagnostic imaging for SPM is a two-view chest X-ray, which has a sensitivity of 50%-90%. When suspicion is high and the chest X-ray is negative, a CT chest without contrast could be the next step in evaluation [8].

Boerhaave’s syndrome is rarely associated with SPM; however, it is important to keep it in consideration due to its associated high mortality rate of 70% [5,7]. This can be suspected in a patient presenting with severe emesis with or without blood, leukocytosis, hypotension, and a prior history of gastroesophageal reflux disease. CT chest without contrast or esophageal swallow imaging can be useful diagnostic tools [8].

As evidenced in our patient, SPM is generally a benign self-limited condition. Management is generally conservative, including rest, oxygen, and analgesia [3-5]. There is no established role for antibiotics unless there is a concomitant infectious process, neither is there a specific follow-up indication for patients who had an uncomplicated SPM. Dajer-Fadel et al. [2] created an algorithm suggesting outpatient follow-up in patients with associated complications (tension pneumothorax, exacerbation, or recurrence). Fortunately, the evolution of this entity is benign with an excellent prognosis and no recurrence in most cases [10].

## Conclusions

Given the benign nature of SPM, it is possibly an underdiagnosed entity. We report this case to increase awareness among physicians of pneumomediastinum as a differential diagnosis in a young male presenting with DKA and dyspnea. Furthermore, we would like to emphasize the benign course of this condition to avoid unnecessary workup and treatment, while providing supportive management.
